# Structural and bio-functional assessment of the postaxillary gland in *Nidirana pleuraden* (Amphibia: Anura: Ranidae)

**DOI:** 10.1186/s40851-020-00160-w

**Published:** 2020-06-05

**Authors:** Yuzhou Gong, Yiwei Zeng, Puyang Zheng, Xun Liao, Feng Xie

**Affiliations:** 1 CAS Key Laboratory of Mountain Ecological Restoration and Bioresource Utilization and Ecological Restoration Biodiversity Conservation Key Laboratory of Sichuan Province, Chengdu Institute of Biology, Chinese Academy of Sciences, No. 9 Section 4, Renmin Nan Road, Chengdu, Sichuan 610041 People’s Republic of China; 2 Universtiy of Chinese Academy of Sciences, 19A Yuquan Road, Beijing, 100049 People’s Republic of China

**Keywords:** Anura, Macrogland, Sexually dimorphic skin gland (SDSG), Specialized mucous gland (SMG), Structure, Ultrastructure, Histochemistry, Behavior, Sexual pheromone

## Abstract

**Background:**

Owing to their incomplete adaptation to the terrestrial environment, amphibians possess complex cutaneous glandular systems. The skin glands not only regulate water loss and respiratory gas and salt exchange, but are also involved in defense against predators and microorganisms, social communication, and reproduction. These glands are distributed throughout the integument, but can accumulate in specific regions, forming visible outgrowths known as macroglands. Some macroglands are sexually dimorphic and mediate intersexual communication and reproductive success. The postaxillary gland is a sexually dimorphic macrogland in *Nidirana pleuraden*. Its biological function and its morphological and histochemical characteristics are unclear. In the present study, we describe the structure and ultrastructure of the postaxillary gland, and explore its main function.

**Results:**

The postaxillary gland has a thinner epidermis than the dorsal region of *N. pleuraden*. In addition to ordinary serous glands (OSG), type I and II mucous gland (I MG & II MG), a type of specialized mucous gland (SMG) is also found to constitute the postaxillary gland. The SMG is larger than other gland types, and consists of high columnar mucocytes with basal nuclei arranged radially toward a lumen. SMGs are positive to periodic acid-Schiff stain and stained blue in Masson’s trichrome stain. A discontinuous myoepithelial sheath lacking innervation encircles SMG mucocytes, and the outlets of such glands are X- or Y-shaped. Transmission electron microscopy reveals abundant secretory granules in SMG, which are biphasic, composed of an electron-opaque outer ring and a less electron-dense core. Lipid droplets, and organelles, such as rough endoplasmic reticulum and Golgi stacks, are located in the supranuclear cytoplasm of the mucocytes in SMG. Female *N. pleuraden* exhibits chemotaxis toward homogenate of the postaxillary gland, but male does not. On treatment with trypsin, this sexual attraction disappears.

**Conclusions:**

The postaxillary gland of *N. pleuraden* is a male-specific macrogland that consists primarily of SMGs, together with OSGs, I MGs and II MGs. Other than their extremely large size, SMGs structurally and histochemically resemble many reported specialized gland types in amphibian sexually dimorphic skin glands. Secretions of the postaxillary gland are proteinaceous sexual pheromones, which are believed to attract females at male calling intermissions.

## Background

As a transitional type of organism which emerged from aquatic environments to inhabit the terrestrial environment during animal evolution, amphibians’ adaptation to subaerial conditions is somewhat incomplete. Their skins, which were embedded with complex and active integumentary apparatuses, play a crucial role in the survival of this taxon. Distinct from other vertebrates, numerous dermal glands are functional in the amphibian skin, which help to regulate respiratory gas, salts, and water exchange [1], and contribute to the discharge of biochemical materials involved in antipredatory [2], antimicrobial [3] and communication activities [4, 5]. Owing to their abundance of cutaneous glands, the skin of Amphibia is capable in maintaining homeostasis, serving as a desirable interface between the animal and its external surroundings.

In the extant amphibians, the skin multicellular glands are simple alveolar and intradermal ones which evolved from single mucocytes and serous cells scattered in the epidermis of their bony fish ancestors [6]. Using morphological (structural and ultrastructural) and histochemical criteria, these cutaneous glands can be categorized into four basic types: serous (granular, venom, or poison), mucous, lipid (wax) and mixed (serous-mucous) glands [7, 8]. Each type of amphibian skin gland exerts certain biological functions: for the serous glands, antipredatory defense [9, 10], nutrient storage [11], antimicrobial activities [12] and chemical communication [13, 14]; for the mucous glands, saline regulation, gas exchange, water loss control, friction reduction [1], immunoreactivity [15], pheromone production [16, 17], amplexus facilitation [18] and parental care [19]; the lipid or wax glands have been observed only in anuran species, their secretions serve to reduce abrasion and dehydration [20]; the mixed glands synergistically produce mucous and serous secretions as an adaptation to environmental and social constraints in urodele larvae [21], but probably represent a transient stage in serous gland development or restoration in anurans [18]. Despite their various functions, all amphibian skin glands involve the integration of four structural components: a myoepithelial sheath, a secretory unit, an intercalated tract (or neck), and a duct [1, 18].

In addition to this microscopic system of gland classification, another common approach to differentiating amphibian glands is based mainly on topographical anatomy, which always involves multiglandular structures that become visible protuberances (patches, swellings, plicae, excrescences, etc.) located on restricted body areas of the animal. The term macrogland has been introduced to describe these macroscopic glandular structures [8]. A macrogland is a cluster of a single glandular type, such as the femoral glands in some mantellid frogs, which are accumulations of serous glands [13], or of multiple kinds of glands, among which stands out a predominant type, such as the parotoid macrogland in *Phyllomedusa* leaf frogs consists of serous, mucous, and lipid glands, but with the serous ones predominate [22]. Microscopic glands that participate in macrogland formation may become more specialized, compared to the ordinary glands which are broadly and randomly distributed in the whole body surface. For example, the mental glands and lateral glands in male *Hypsiboas punctatus* contain both specialized serous glands and specialized mucous glands which manifest incongruent cytology and staining properties with the ordinary serous and mucous glands [23]. The term “specialized” often refers to localized glands that carry out particular functions. They are believed to actively engage in antipredatory defense, social communication, and reproductive mechanisms in both Anura [24] and Urodela [25].

Amphibian macroglands used as arsenals of toxins in chemical defense against predators have been broadly investigated, including the parotoid glands in bufonids, pelobatids, ambystomatids, plethodontids, and salamandrids [10, 18, 26, 27]; the tibial glands in *Limnodynastes* and *Gephyromantis* [2, 28]; the leg glands in *Bombina* [29]; the inguinal glands in *Physalaemus* [30, 31]; the supratympanic glands in hylids, pelodryadids, and phyllomedusids [9, 32, 33]; and the caudal glands in *Hydromantes* and *Triturus* [34, 35]. In the case of macroglands that mediate amphibian social communication and reproductive patterns, they are roughly equivalent to “sexually dimorphic skin glands” (SDSG), a term proposed by Thomas et al. [7] to extend the definition of the preexisting word “breeding glands”. Many SDSGs have been proved or speculated to intervene in intraspecific communication and reproduction. For instance, the nuptial pads that develop on male amphibians’ thumbs or limbs help them to fasten their grip during amplexus [36], and may also transmit pheromones to females through skin abrasion [37]; the abdominal or pectoral glands in microhylids secrete a glue-like material to facilitate male–female pair formation [38]; the abdominal, dorsal, and cheek glands in salamandrids [4, 39–41], the mental glands in plethodontids and hylids [23, 25, 42], the femoral glands in mantellids [14, 43], the gular glands in hyperoliids [44], the iliac glands in Cycloramphus [45] and the postaxillary glands in *Hymenochirus* [46] are believed to produce pheromones that alter the behavior of conspecific female or male. Notably, some SDSGs are not macroglandular structures [1, 7], and some amphibian reproductive macroglands are not sexually dimorphic [5].

*Nidirana pleuraden*, the Yunnan pond frog [47] or Yunnan music frog [48], is endemic to Southwestern China and inhabits paddy fields, ditches, and ponds at elevations from 1150 to 2300 m asl. on the Yunan–Guizhou subtropical plateau. Male *N. pleuraden* possesses a pair of subgular external vocal sacs, and during the breeding season (June to July) [47], it develops a pair of male-specific postaxillary glands. The SDSG, postaxillary glands, have been documented in several anuran amphibians as round or irregular-shaped cutaneous swellings lying behind base of forelimb, colored pale brown (in *Nidirana*) or whitish (in *Hymenochirus*) [7, 46, 48]. The postaxillary glands in a congeneric species of *N. pleuraden*, east China music frog (*N. adenopleura*) have been examined structurally and histochemically to determine their similarity to the nuptial pads in *Xenopus laevis* [7], whereas the postaxillary glands in *Hymenochirus* have been demonstrated ethologically to be the sole source of secreted sex attractants [46]. Nonetheless, there have been no comprehensive studies of the morphology and biological function of the postaxillary glands in Anura, especially with respect to the situation that most attention to SDSGs has been given to the nuptial pads [18]. By integrating morphological, histochemical, and ethological methods, we seek to describe the structure and ultrastructure of the postaxillary gland, determine the nature of this macrogland, explore its main function, and the likely interaction between its secretions and auditory signals in *N. pleuraden*.

## Results

### Macroscopic structure of the postaxillary glands in *N. pleuraden*

**Fig. 1:**
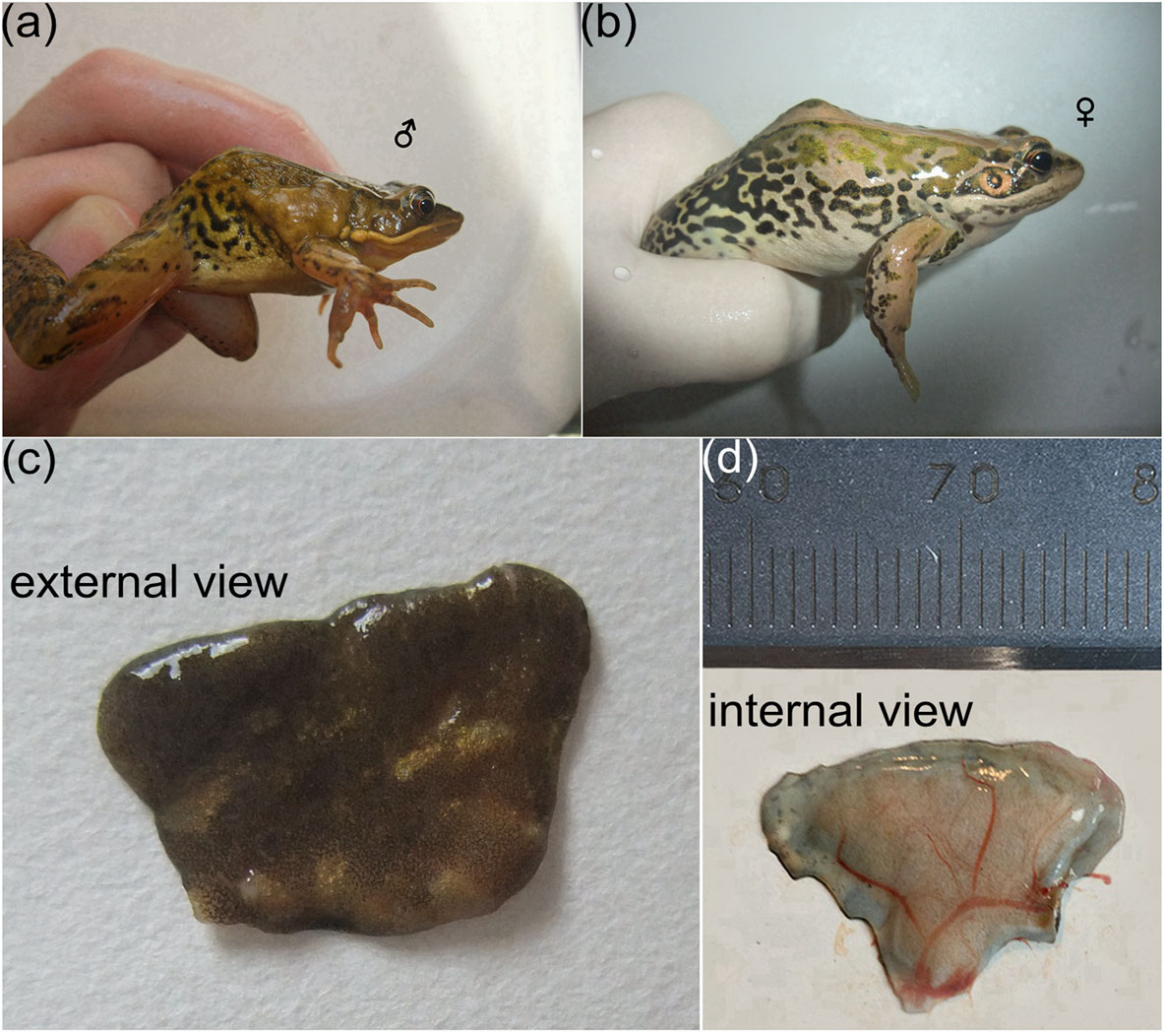
Macroscopic view of the postaxillary gland in *N. pleuraden*. **a** Lateral view of a male *N. pleuraden*. **b** Lateral view of a female *N. pleuraden*, note the absence of postaxillary gland. **c** A freshly dissected postaxillary gland. **d** The inner side of a freshly dissected postaxillary gland

The postaxillary glands are male-specific, comprising a pair of well-defined and enlarged skin macroglandular structures in *N. pleuraden*, which lie behind bases of the forelimbs (Fig. 1a, b, c). These may extend backwards to the anterior end of the ilium, become thinner and eventually end in the front half of the dorsolateral regions. Surfaces of the postaxillary glands in *N. pleuraden* are normally rough and granular, which differ from the smooth surfaces described in other *Nidirana* species [48]. Visually, the postaxillary glands are yellowish brown or pale brown with dark brown or black irregular spots (Fig. 1a, c). The postaxillary gland gradually regresses in the weeks after breeding (see Additional file 1, Fig. S1). Inspecting a freshly excised postaxillary gland, abundant blood vessels and capillaries are present on the inner side (Fig. 1d). The average width, height, and thickness of the 14 postaxillary glands we collected from seven euthanized male frogs was 12.07 ± 0.37 mm (10.22–14.54 mm), 7.57 ± 0.29 mm (6.10–9.34 mm) and 1.35 ± 0.01 mm (1.28–1.46 mm), respectively.

### Structural and histochemical observations

**Fig. 2:**
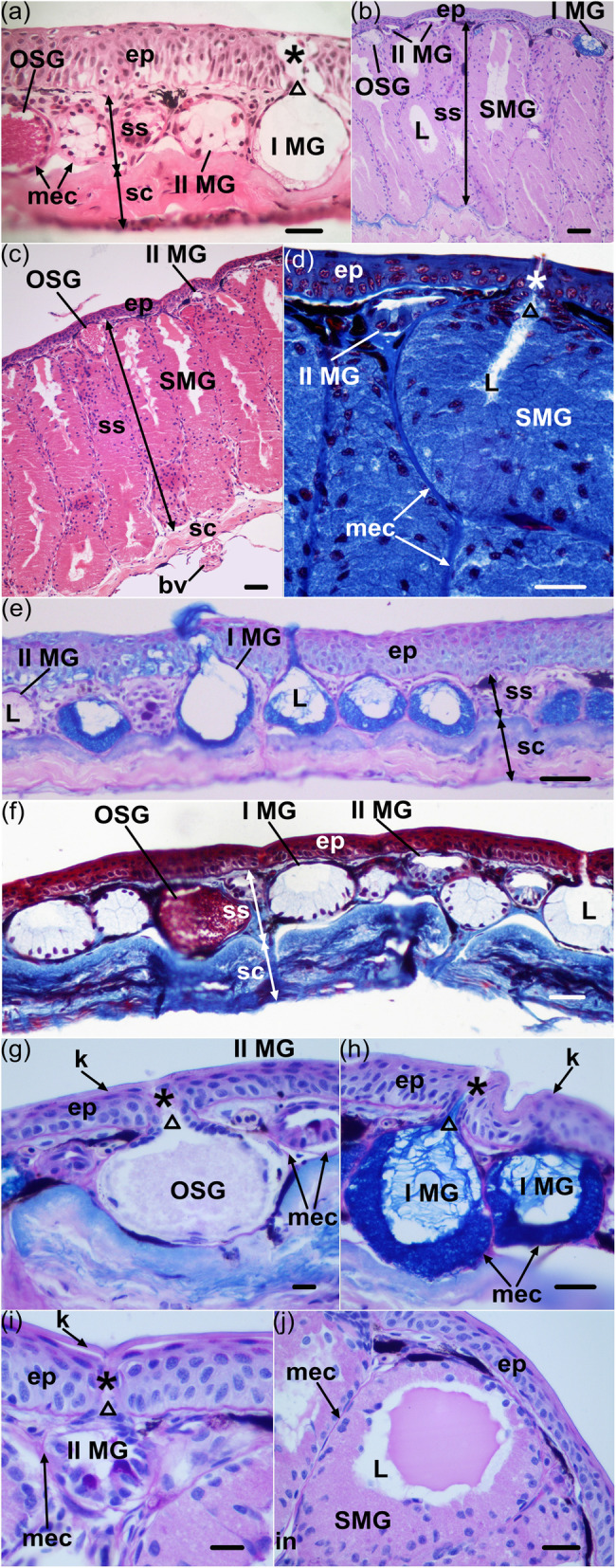
Structural and histochemical characteristics of dorsal skins and postaxillary glands of male *N. pleuraden*. **a** HE staining of the dorsal skin. **b** AB-PAS staining of the postaxillary gland. **c** HE staining of the postaxillary gland. **d** Masson’s trichrome staining of the postaxillary gland. **e** AB-PAS staining of the dorsal skin. **f** Masson's trichrome staining of the dorsal skin. **g** Magnified view of ordinary serous glands in dorsal skins stained with AB-PAS method. **h** Magnified view of the type I mucous glands in dorsal skins stained with AB-PAS method. **i** Magnified view of the type II mucous glands in postaxillary glands stained with AB-PAS method. **j** Magnified view of a lumen contains secretory materials of the specialized mucous glands in postaxillary glands stained with AB-PAS. ep, epidermis; ss, *stratum spongiosum*; sc, *stratum compactum*; mec, myoepithelial cell; I MG, type I mucous gland; II MG, type II mucous gland; OSG, ordinary serous gland; SMG, specialized mucous gland; bv, blood vessel; L, lumen; *, gland duct; △, gland neck; k, keratinized epidermal cell; in, interstice. Bars = 100 μm (**b**, **c**, **e**, **f**), 50 μm (**a**, **d**, **h**, **j**) and 25 μm (**g**, **i**)

Comparing the transverse sections of the postaxillary gland with those of the dorsal skin, we found that they share the usual structure in anurans: A layer of keratinized cells covers the epidermis, below which is the dermis. The epidermis is composed of 4–5 cell layers, the 1–2 layers of squamous cells, which are more superficial, and the 3–4 layers of cubical cells, below. The dermis consists of the *stratum spongiosum*, which is penetrated by numerous simple alveolar glands (Fig. 2a–f), and the *stratum compactum*, under which blood vessels are enriched (Fig. 2c).

Although the postaxillary gland and male skin of dorsal regions are similar in basic structure, there are some disparities between them. The postaxillary gland is a swollen skin apparatus macroscopically, and is approximately four times thicker than the dorsal skin (compare Fig. 2b–d to Fig. 2a, e, f). The hypertrophy of this macroglandular structure is mainly ascribed to the thickening of the dermal parts, whereas its epidermis (68.07 ± 3.69 mm, *n* = 10) is much thinner than that in the dorsal skin (102.13 ± 6.86 mm, *n* = 10, Wilcoxon rank-sum test: *Z* = − 3.52, *P* < 0.01). Most tellingly, we recognized four types of simple glands within the *stratum spongiosum*, which were ordinary serous glands (OSGs), type I mucous glands (I MGs), type II mucous glands (II MGs) and specialized mucous glands (SMGs), according to their morphologies and histochemical properties, and the SMGs were located in the postaxillary glands exclusively (Fig. 2b–d, j).

The four types of glands identified from male dorsal skins and the postaxillary glands possess a basic structure: an intraepidermal duct, a neck, and a secretory unit surround by a layer of myoepithelial cells (Fig. 2d, g-i). There are distinct features in morphology or histochemistry of each gland type. To be distinguished clearly from the mucous glands, the ordinary serous glands own syncytial secretory units which contain discrete eosinophilic granules (Fig. 2a, c). The spherical secretory granules in OSGs are barely stained with PAS or AB pH 2.5 (Fig. 2b, g), but stained scarlet in Masson’s trichrome, indicating a compact structure not formed by mucopolysaccharides.

The I MGs, which are in spherical shapes and formed by a single layer of moderately tall prismatic secretory cells with basal nuclei, are more numerous in male dorsal skin than in the postaxillary glands,. The mucocytes surrounding an evident lumen decrease in height from the glandular bottom towards the neck (as clearly demonstrated in the Masson’s trichrome staining, Fig. 2f), basal epithelium of the neck appears to be continuous with flattened cells of the gland duct. When stained with HE or Masson’s trichrome methods, the prominent lumina of I MGs are quite empty, whereas they show strongly positive reaction to AB pH 2.5 by presenting copious unstructured floccules (Fig. 2b, e, h). The blue stains of I MG suggest acidic mucosubstances (carboxylated acidic glycosaminoglycans and acidic glycoproteins) are replete both in the cytoplasm of the mucocytes and the glandular lumina. The other type of ordinary mucous glands, II MGs are quite distinct from the I MGs; these elliptic glands distribute in dermis of the postaxillary glands as frequently as in the dorsal regions. A relatively exiguous lumen is encompassed by low cuboidal secretory cells with middle or basal nuclei (Fig. 2b, d, f). The mucocytes become flat, forming an imbricated squamous epithelium at the glandular neck. Normally, the lumina of II MGs show a state of devoid secretions in all the three histochemical staining methods that we use, yet the cytoplasms of their mucocytes are metachromatically stained with PAS (Fig. 2a–f), the apical parts of some mucocytes are violet while the rest parts and most other mucocytes are in light purple (Fig. 2i), which implies the possible syntheses of neutral carbohydrates to acidic mucopolysaccharides or glycoproteins in II MGs.

Besides the two types of ordinary mucous glands, further secretory units are predominant and found only in the postaxillary glands, which can clearly be related to the mucous type due to their organization and secretion. The term “specialized mucous glands” was used to describe them. The SMGs are rod-like and much larger than the ordinary ones both in secretory cells and glandular height (one-way ANOVA: F = 8828.93, *P* < 0.0001 and F = 768.51, *P* < 0.0001) and consist of high columnar mucocytes usually with basal nuclei arranged radially toward the inner lumina (Fig. 2b-d). Mucocytes near the neck area become low to moderately tall and imbricate to form a stratified structure beneath the glandular duct (Fig. 2d). There are wide interstices between the myoepithelial sheaths of some SMGs (Fig. 2j). Dense, eosinophilic materials fill their cytoplasm and sometimes in fluid or mucous phases in their lumina (Fig. 2c). The cytoplasmic and luminal contents are positive to PAS (Fig. 2j), indicating the presence of neutral carbohydrates or mucoproteins. With the Masson’s trichrome stain, the gland products turn dark blue (Fig. 2d), a characteristic color of collagen fibers or proteinaceous substances. Morphological and histochemical traits of the four types of alveolar glands that we found in the paraffin sections of male dorsal skins and the postaxillary glands are compared in Table 1.

**Table 1:** Morphological and histochemical characteristics of the four types of simple alveolar glands in *N. pleuraden*

	I MGs	II MGs	SMGs	OSGs
Topological distribution	Dorsal skins & postaxillary glands	Dorsal skins & postaxillary glands	Postaxillary glands exclusively	Dorsal skins & postaxillary glands
Height of mucocyte (μm)	37.21 ± 0.57^a^	20.51 ± 0.54^b^	112.64 ± 0.46^c^	\
Height of gland (μm)	112.84 ± 13.63^a^	78.13 ± 6.70^b^	691.99 ± 12.54^c^	141.34 ± 7.09^a^
Reaction to AB-PAS staining	Stains blue	Stains light purple to violet	Stains light purple	Feebly tinged with pink
Reaction to Masson’s trichrome staining	Feebly tinged with blue	Stains dark blue	Stains dark blue	Stains dark scarlet

If the superscripts beside values are same, the disparity in a row has no statistical significance, otherwise the difference is statistically significant (one-way ANOVA plus Bonferroni-adjusted LSD post-hoc test)

### Ultrastructural observations

**Fig. 3:**
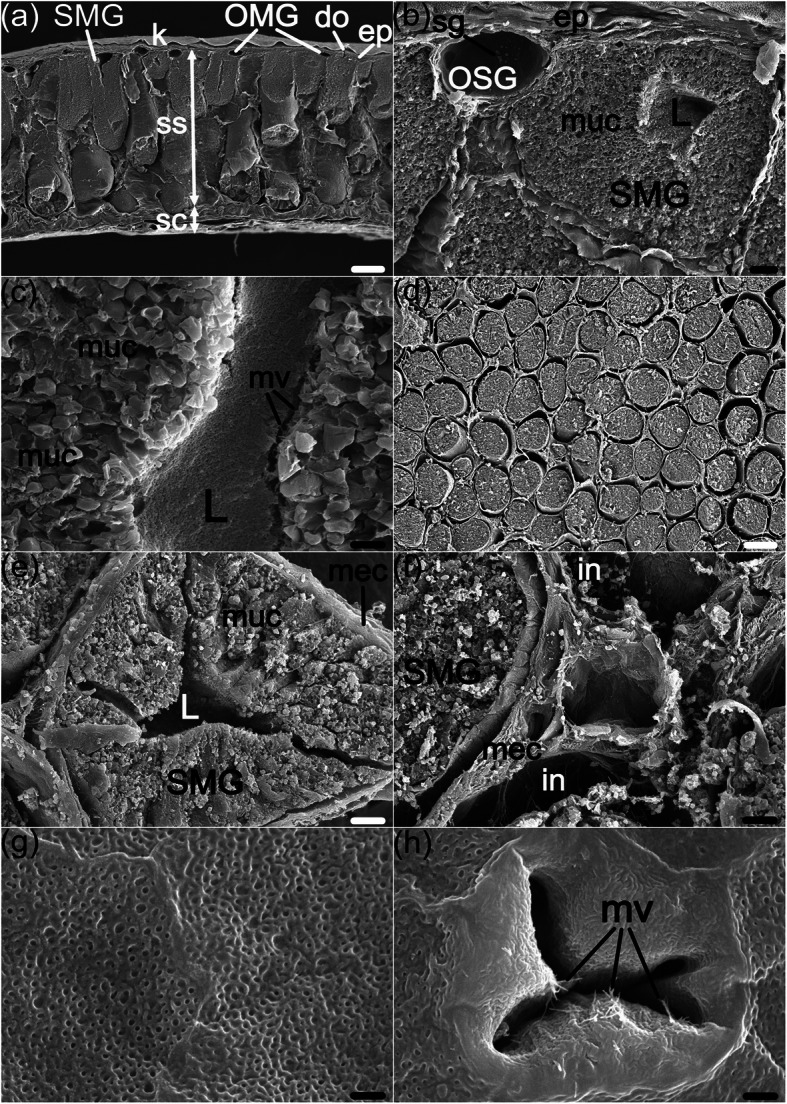
Scanning electron micrographs of the postaxillary glands in male *N. pleuraden*. **a** Transverse section of the postaxillary gland. **b** Higher magnification image of specific regions in (**a**), showing an OSG and a SMG. **c** Mucocytes surround an obvious lumen in SMGs. **d** Longitudinal section of the postaxillary gland. **e** Longitudinal section of a SMG, its lumen is surrounded by numerous mucocytes and the mucocytes are all encircled by the myoepithelial sheath. **f** A complex, irregular connective structure locates at the interstice between myoepithelial sheaths. **g** Normal texture of the skin surface of the postaxillary gland. **h** A close-up of the glandular outlet, note the intraductal microvilli. ep, epidermis; do, duct opening; SMG, specialized mucous gland; OMG, ordinary mucous gland; OSG, ordinary serous gland; sg, secretory granule; ss, *stratum spongiosum*; sc, *stratum compactum*; muc, mucocyte; mec, myoepithelial cell; L, lumen; mv, microvilli; in, interstice. Bars = 500 μm (**a**, **d**), 50 μm (**b**, **e**, **f**), 10 μm (**c**) and 5 μm (**g**, **h**)

The descriptions of ultrastructure are specific to the postaxillary glands, as we did not examine the skin of other body regions under SEM or TEM. When inspected by SEM, the transverse sections of the postaxillary glands show similar structural patterns to the results with LM (Fig. 3a). However, the outer skin layer with minute papillae formed by keratinized epidermal cells is more conspicuous than in LM (Fig. 3b). The two types of ordinary mucous glands (I MGs and II MGs) are difficult to distinguish from each other reliably on account of their similar glandular profiles and both having lumina devoid of secretions. OSGs could be distinguished due to the presentation of spherical secretory granules in their lumina (Fig. 3b). SMGs are recognized with ease, as the entire postaxillary gland is filled with rod-like glands (Fig. 3a, d).

The high columnar mucocytes surrounding the glandular lumen are in tight reciprocal adhesions, which imports difficulties in telling their exact cellular boundaries (Fig. 3b, c, e). The apical poles of these cells are covered by tufts of microvilli, which render a rough appearance to the luminal surface. Resembling to I MGs and II MGs, lumina of SMGs are scarce of secretory materials, whereas the most intracytoplasmic spaces of mucocytes are occupied by polyhedral granules (Fig. 3c).

When dissected longitudinally, the postaxillary glands emerge a honeycomb-like arrangement (Fig. 3d). The round components are closely-packed SMGs. There are wide interstices between the bases of mucocytes and the glandular sheaths consisted of myoepithelial cells, and the in-between rooms of myoepithelial sheaths encircling adjacent glands may be empty or inhabit rather complex structures (Fig. 3f). Surfaces of the postaxillary glands are rough, full of pores and wormlike veins under a large magnification of the SEM (Fig. 3g). X- or Y-shaped duct openings are widely distributed in the same region; they resemble stomata in the superficial epidermal layer, and distinct from the common skin texture, the epithelium that lines the outlet is smooth (Fig. 3h). The duct opening slightly protrudes from the epidermis and shows inner microvilli, which we deem a characteristic trait of the apices in SMGs’ mucocytes.

**Fig. 4:**
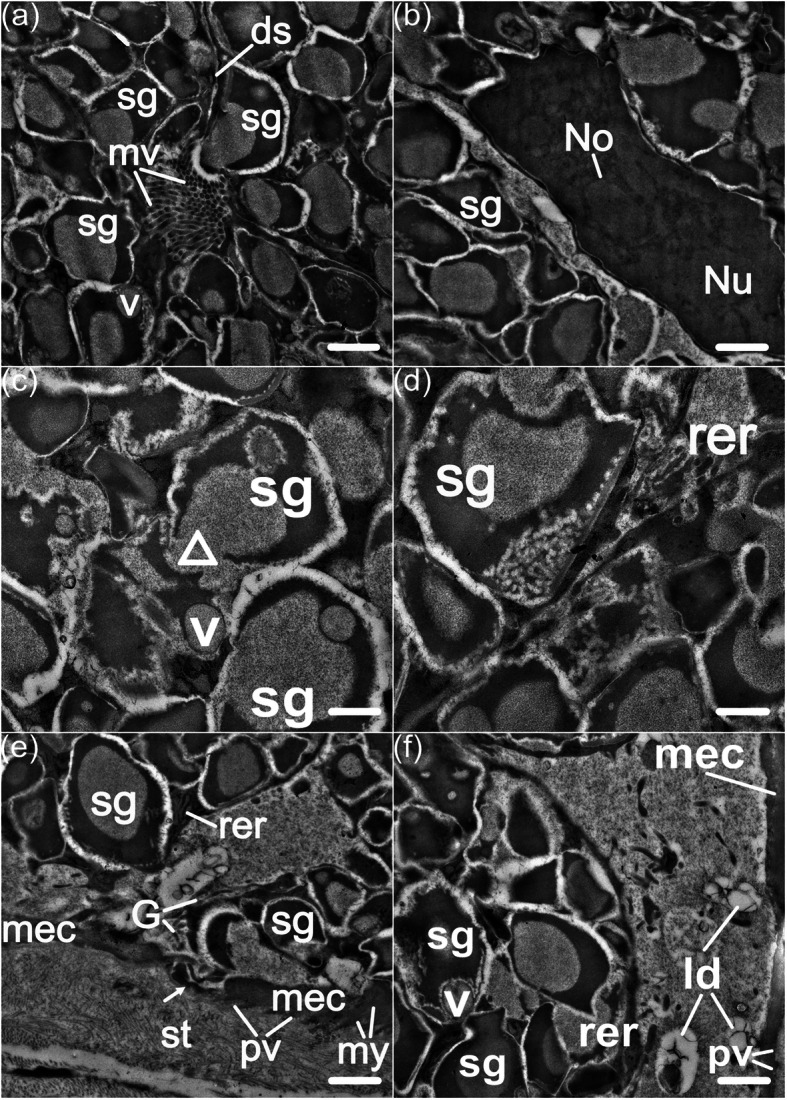
Transmission electron micrographs of SMGs in the postaxillary glands of male *N. pleuraden*. **a** Longitudinal section of SMGs, note the microvilli and the desmosome connecting two contiguous mucocytes. **b** A euchromatic nucleus surrounded by numerous secretory granules. **c** Translucent vesicles are released from large secretory granules via a breach on granular border. **d** Secretory granules hold materials of varying electron-density, note the dilated rough endoplasmic reticulum at the supranuclear region. **e** The discontinuous myoepithelial sheath across the secretory unit and glandular stroma, note the plasmalemmal projection of the mucocytes (arrow). **f** supranuclear cytoplasm of the mucocyte contains lipid droplets in low electron-opaque. sg, secretory granule; mv, microvilli; ds, desmosome; rer, rough endoplasmic reticulum; v, vesicle; No, nucleolus; Nu, nucleus; △, breach of the secretory granule; G, Golgi stack; mec, myoepithelial cell; my, myofilament; st, stroma; pv, pinocytotic vesicle; ld, lipid droplet. Bars = 1 μm (**a**, **b**, **e**, **f**) and 0.5 μm (**c**, **d**)

The most salient structures that we found in SMGs under TEM exanimations are large secretory granules within their mucocytes (Fig. 4a). These granules are so numerous that they appeared wedged into one another, resulting in polyhedral or irregular shapes. They are biphasic, with heterogeneous composition, and normally appear as an electron-opaque outer ring and a less electron-dense core (Fig. 4). Some moderately electron-opaque vesicles traverse the outer ring of the secretory granule (Fig. 4c, f), which may represent an intracytoplasmic process of condensation or fusion. The apical microvilli of mucocytes that we observed under SEM are shown as regional agglomerations of electron-opaque dots here due to the longitudinal sections of SMGs; contiguous mucocytes are joined by desmosomes (Fig. 4a). Corresponding to our SEM observations, the cytoplasm between secretory granules and nuclei is rather narrow, and the nuclei appear euchromatic and in irregular outlines, nucleolus is found sometimes (Fig. 4b).

The thin myoepithelial sheath surrounding the secretory unit is discontinuous and lacks any direct nerve support, which contains plentiful myofilaments orderly distributed along the longitudinal cellular axis (Fig. 4e). Above the myoepithelial cell layer is the supranuclear cytoplasm, where the regular or dilated cisternae of rough endoplasmic reticulum and Golgi stacks, as well as secretory vesicles are located (Fig. 4d–f). The breach on the myoepithelial sheath allows the mucocytes to reach underlying glandular stroma through their basal plasmalemmal projections (Fig. 4e). Lucent pinocytotic vesicles are found at both sides of the myoepithelial sheath, whereas larger elliptical vesicles that hold low electron-dense and structureless materials, resembling lipid droplets, are only situated against the myoepithelial cell border facing mucocytes (Fig. 4e, f).

### Animal behavior tests

In the five sets of behavior tests we performed, only one animal preference was detected, which is female *N. pleuraden* prefers to stay at the tank end positioning postaxillary gland stimulus (436.80 ± 69.19 s) rather than remain at the distilled water end (163.20 ± 69.19 s, Z = 2.37, *P* < 0.05), and when exposure to male advertisement calls, this preference disappears (350.90 ± 79.70 s vs. 249.10 ± 79.70 s, Z = 0.88, *P* = 0.38), likewise adding trypsin solution to react with the homogenate of postaxillary glands (308.60 ± 89.67 s vs. 291.40 ± 89.67 s, Z = 0.20, *P* = 0.84). Neither females select between the homogenate of male dorsal skins (318.60 ± 79.17 s) and the distilled water (281.40 ± 79.17 s, pooled t-test, t = 0.33, *P* = 0.74), nor males show predilections to the homogenate of postaxillary glands (321.50 ± 79.77 s) or distilled water (278.50 ± 79.77 s, t = 0.38, *P* = 0.71).

**Fig. 5:**
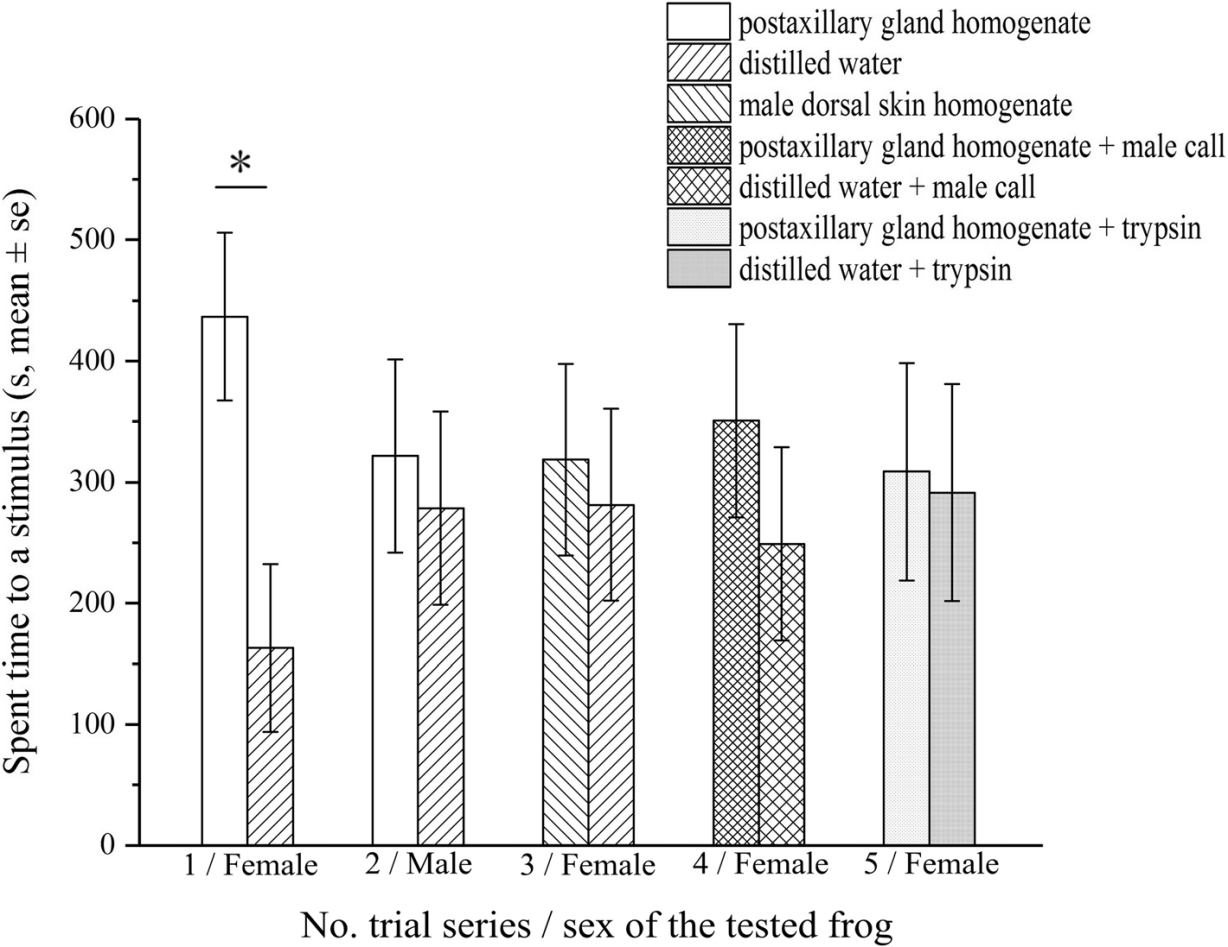
Results of the five series of animal behavior trials. Note only females show a preference of homogenate of the postaxillary glands over distilled water. Trial series 1, female chooses between homogenate of the postaxillary gland and distilled water; trial series 2, male chooses between homogenate of the postaxillary gland and distilled water; trial series 3, female chooses between homogenate of male dorsal skins and distilled water; trial series 4, female chooses between (homogenate of the postaxillary gland plus male advertisement call) and (distilled water plus male advertisement call); trial series 5, female chooses between trypsin treated homogenate of the postaxillary gland and trypsin treated distilled water

Noticeably, in the 10 trials of females choosing from postaxillary glands homogenate and distilled water, seven of the 10 tested frogs swam to the postaxillary glands end immediately after the acclimation period, and eight frogs were stationary at the postaxillary glands end when the trial was finished, this kind of bias was rarely seen in other trial sets. Results of the behavior tests are illustrated in Fig. 5.

## Discussion

To date, most studies of anuran SDSGs have focused on nuptial pads [7, 18, 37]. In the present study, we report the first integrative approach, including morphological (structure and ultrastructure), histochemical and ethological methods, to examine the postaxillary gland, another sexually dimorphic macrogland which prevails in *Hymenochirus*, *Pseudhymenochirus* [49] and *Nidirana* [47, 48].

Unsurprisingly, the postaxillary glands of *N. pleuraden* are composed of specialized mucous glands, besides ordinary serous glands and mucous glands. What we did not foresee was the extremely large volumes of the SMGs, which predominated in the postaxillary glands; these were 692 μm high in average, much larger than any known anuran SMGs—the size of mental glands’ SMGs in hylids and hyperoliids is normally 245 to 260 μm, with a maximum to 576 μm in *Hyloscirtus bogotensis* [23]. The ordinary serous glands distributed both at the male dorsal skins and the postaxillary glands, they showed typical morphological and histochemical characteristics mentioned in other amphibians, for having relatively thick myoepithelial cells layer, eosinophilic contains and negative to AB pH 2.5 or PAS stains [1, 9], no sign of specialization of the serous kind was detected among histological sections.

Like the topological distribution of OSGs, two kinds of ordinary mucous glands were identified from dorsal skins and the postaxillary glands. The subdivision of type I mucous glands and type II mucous glands was mainly based on the morphologies of their constituent secretory cells (moderately tall prismatic mucocytes in I MGs and low cuboidal mucocytes in II MGs) and histochemical responses of the luminal or intracytoplasmic secretions (intensely positive to AB pH 2.5 in I MGs and positive to PAS in II MGs). Our observations on the two distinct ordinary mucous gland types correspond to the previous reports that demonstrated I MGs and II MGs exist in the skin of *Rana amurensis* [50], and the dorsal skin and dorsolateral fold of *Sylvirana latouchii* [51]. Antoniazzi et al. [22] also found two kinds of ordinary mucous glands locate at the dorsal skin of *P. distincta*, but the I MGs they examined were positive to the PAS staining instead of AB pH 2.5, whereas their II MGs were stained with both PAS and AB pH 2.5, implying variations in secretions of ordinary mucous glands between anuran species.

When compared to the serous glands which act as antipredatory arsenals [10, 27, 34], specialization of the other gland types, especially link to the mucous glands, which are the most widespread units throughout amphibian skins and usually participate in fundamental physiological activities, drew much less attention. The apparent “least specialized” glands have often been overlooked until they were related to mental glands in some plethodontids [52]. Since then, many amphibian SDSGs, which play important roles in reproductive activities or parental cares, have been suggested a mucous gland nature [1, 7, 17, 53]. The postaxillary gland of *N. pleuraden* consisted of derived mucous glands primarily under our LM, SEM and TEM observations, was analogous to most discovered SDSGs, except the femoral glands in Mantellidae (compose of specialized serous glands (SSGs), [13]), the iliac glands in *Cycloramphus* (contains mainly SSGs, [45]), and the mental and lateral glands in *Hypsiboas punctatus* (both SMGs and SSGs present, [23]). High columnar adenocytes radially arranged around an obvious lumen and contained abundant secretory granules in their cytoplasms were the essential morphological features of the SMGs in the postaxillary gland, which in consensus with the descriptions about other amphibian SMGs [1, 17, 23]. The myoepithelial sheaths enveloping the secretory units of SMGs were relatively thin and discontinuous, which represented the type I myoepithelial cell layer that commonly encircle SMGs’ mucocytes in other amphibians [54]. Noticeably, direct innervations were not witnessed in this contractile apparatus, unlike the situations have been reported in OSGs and SSGs in other anuran species [9, 10, 23], and SMGs in the dorsal and mental glands of some urodeles [1], indicating a merocrine release process and a relatively low degree of functional specialization of SMGs in the postaxillary glands. The cutaneous glandular duct openings of SMGs had a rather smooth surface comparing to the pitted superficial keratinized layer of the epidermis, which resembled the type I outlets occur in mental and lateral glands of male *Hypsiboas punctatus* [23].

Histochemically, SMGs in the postaxillary glands reacted positively to PAS stain, like most other SMGs in different species, with one exception that the SMGs of *R. iberica* show no response to PAS [1]. It was noteworthy that the pervasive polyhedral secretory granules in the cytoplasm of SMGs’ mucocyte have two subunits of different electron-densities. Secretory granules of other SMGs are normally homogenous, as they may possess different electron-opaques along the axis from the basal supranuclear cytoplasm to apical luminal border in mucocytes, but not within a single granule [23]. Similar cases were found in SMGs of *Hypsiboas punctatus*, and more frequently, in serous glands [10, 23]. It has been proposed that granules mainly consisting of protein are always dense and have internal subunits, besides we did not detect any glycogen particles, but rough endoplasmic reticulum and Golgi stacks in the supranuclear regions of the mucocytes in SMGs, the positive reaction to PAS and Masson's trichrome stains may be interpreted as biosynthesis and storage of neutral mucoproteins. In addition, some electron translucent vesicles occurred in the basal subnuclear cytoplasm of the mucocytes represented lipid droplets [55], which are usually found in OSGs [23, 32, 56, 57] or lipid glands [58] rather than in SMGs, were possibly residual molecules from the steroid metabolism of pheromones production or carriers of pheromone precursors [1, 9]. Although the typical organelle concerning lipid biosynthesis, smooth endoplasmic reticulum was never observed in the cytoplasm, it probably ascribed to the large quantities of electron-opaque secretory granules filled in mucocytes.

According to the structural, ultrastructural and histochemical traits in SMGs, they could be easily distinguished from OSGs and OMGs. Even though there were evidences on OMGs transforming into SMGs after testosterone injection in plethodontids [53], we do not reckon the SMGs and the OMGs in *N. pleuraden* stand for one glandular type in different stages of maturation or asynchronous secretory phases caused by local fluctuation, considering the SMGs’ specific topological affinity to the postaxillary glands.

Compared with distilled water, female *N. pleuraden* prefers the homogenate of male postaxillary glands. However, male frog is neither attracted nor repelled by the homogenate of the postaxillary gland. Taken together, these demonstrate the secretions of the postaxillary gland effect as sex attractant rather than aggregation or territorial pheromone [59]. Thinning of the epidermis on the postaxillary regions may also facilitate attractant release [22]. Female has no partiality for the homogenate of male dorsal skin suggests that the manufacture of attractant is restricted to SMGs, which coincides with the histochemical and ultrastructural observations. Unlike volatile alcohols or macrolides secreted by the femoral gland in Mantellid frogs [14, 43], we deduce that the nature of the attractant herein is water-soluble peptides or proteins, as trypsin treatment eliminates the sexual appeal of the postaxillary gland homogenate. When male advertisement calls are also provided as stimuli, female shows hesitation on selecting homogenate of the postaxillary gland over distilled water, probably means that acoustic signals predominate in the context of the species’ reproductive communications, which in concert with many experimental proofs that acoustic cues alone, rather than any other signal form are sufficient to attract conspecifics in Anura [44, 60, 61].

Many SDSGs have been proved to involve in pheromone productions in amphibians, regardless of urodelean species [4, 16, 39, 41, 62] or anuran species [13, 14, 43, 45, 46]. In some ranid species (*R. dalmatina*, *R. iberica* and *R. italic*), the SDSGs are diffused in the male dorsal skins rather than segregate in macroglandular structures [1], and the vocal sacs are disappeared in these three anurans. Since vocalization plays a substantial role in attracting females as well as announcing occupied territory to other males, Brizzi et al. [1] proposed that the lack was a secondary loss, and the production of chemical signals would be an evolution of alternative communicational strategies. This hypothesis may be applicable in some European mute *Rana* species, but when we take a glance at a broader scenario, it becomes less suitable since anurans who communicate with acoustic signals also possess SDSGs have been documented several times [13, 14, 43, 44, 46, 61], so is the case in *N. pleuraden* [47].

It is widely accepted that chemical cues are the salamander counterpart of auditory vocalization in frogs and toads [25], thus a theory stating SDSGs or breeding glands in modern anurans may simply be vestigial traits that reflect dependence on chemosignals prior to the evolution of acoustic systems seems to be quite plausible [61, 63]. For instance, in *Leiopelma hamiltoni*, a primitive anuran lineage, calling was not observed, but chemicals played a fundamental role in its social communications [63]. Because *N. pleuraden* is a relatively advanced species of Neobatrachia [47], and the present study has demonstrated that its postaxillary glands’ secretions can attract females solely, we do not in favor of such a theory. In contrast, we suggest that chemical communication is ubiquitous in anurans, although its significance remains underappreciated.

It has been reported that in *Pseudophryne bibronii*, male chemosignals combined with acoustic signals improve the ability of females to find nest sites [61], and a more recent study showed multiple kinds of modern anuran species transcribe sodefrin precursor-like factors (a well-studied pheromone system in Urodela) related genes in their SDSGs [42]. Chemical mate attractants have probably been maintained or evolved in these anuran species due to other constraints on intersexual communication. Evidence shows that impaired visual communication impels the use of chemosignals in animals [46], which tallies with situations in the breeding environment of *N. pleuraden*. The frog is nocturnal and hides in very dense hydrophytes during the breeding season (see Additional file 1, Fig. S2), in a condition which acoustic signals may also susceptible to diffuse [64]. As challenged by such ecological restraints, it is not surprising the behavior of animals is mediated by a multisensory system (acoustic, chemical, and possibly visual signals).

Amphibian sexual pheromones are involved in mate location [4, 5, 46], evaluation [61] and copulatory synchronization [16, 49, 65]. Observations of the mental and lateral glands in Cophomantini (a hylid tribe) suggest the locations of SDSGs are closely related to the forms of physical contact during amplexus [57], like male plethodontid salamanders using the hypertrophied premaxillary teeth to abrade the female’s skin and vaccinating secretions from the mental glands into the abraded site [25] and similar functions of the nuptial pads in *R. temporaria* [37]. In these cases, courtship pheromones alter female’s mating behavior must happen afterwards initial contact. However, the postaxillary regions of *N. pleuraden* do not come into direct contact with females in amplexus, thus the bio-function of the postaxillary gland should intervene in the species’ reproduction before pair formation. We speculate that the secretions of the postaxillary gland assist female frogs in mate localization when a solicitous male is in calling intermissions, and its efficacy is overridden by advertisement calls. Suffice it to say, the acoustical and chemical signals have different applicable scenarios.

In light of that acoustic signals transmit quickly but are expensive to produce and bear short-lived messages, while chemosignals are cheap to produce and provide long-lasting messages but have the disadvantages of the speed and range of transmission, combining both channels in one’s communications increase the efficacy of message transfer undoubtedly. If the chemosignals biosynthesized and released by the SDSGs not only save energy, as well as bringing down the possibility of attracting predators in a courting male [66], but also benefit females in reducing mate search costs, this chemosignal generating and sensory intersexual system in Anura would has been honed over evolutionary time, alike the scenario in Urodela and Apoda [67], or the vocalization and auditory system in itself.

Last but not least, we do not exclude the possibility that the secretions of the postaxillary gland bear information on the quality or genetic relatedness of the male frog, and female identifies and localizes a preferred one after evaluating these chemosignals emitted from a chorus of males. More elaborately designed experiments are required to reveal the compelling function of the postaxillary gland in *N. pleuraden*. Further researches should also focus on identifying and separate the specific component secreted by the postaxillary gland, leading new perspectives of pheromonal communication in Anura.

## Conclusions

The present study examines the structure, histochemical characteristic and bio-function of the postaxillary gland in *Nidirana pleuraden*. Besides the ordinary types of serous gland and mucous gland, a specialized form of mucous gland is found exclusively in this sexually dimorphic macrogland. The specialized mucous gland (SMG) is much larger than other glands, its high columnar mucocytes have basal nuclei, arranging radially toward an obvious central lumen. What inside the lumen and cytoplasm of the mucocytes is eosinophilic, positive to periodic acid-Schiff stain, and stained blue with Masson's trichrome. A discontinuous myoepithelial sheath without direct nerve supply is observed to surround mucocytes of SMG, as well as the X- or Y-shaped glandular outlets distributing at the superficial epidermis. Secretory granules in SMG appear to be biphasic, reveal as a less electron-dense core encircled by an electron-dense outer ring. In addition to numerous secretory granules, lipid droplets, and organelles, such as rough endoplasmic reticulum, Golgi stacks are also detected in the mucocytes. The homogenate of the postaxillary gland attracts female *N. pleuraden*, but has no attracting or repelling effect to males. And if introduces male advertisement call, or trypsin treatment, the sexual appeal vanishes. Taken together, we think the postaxillary gland plays a key role in the specie’s reproduction: its secretions are proteinaceous sexual pheromone, which facilitates females to locate males when they cease calling. Moreover, this study provides new evidences to the field of anuran chemical communication, which is on early stages of development with limited cases. Knowledge on the topological distribution, structural diversity and histochemical variance of sexually dimorphic skin glands in an enlarged phylogenetic scenario, likewise the accurate identification of the glandular secretions would help to elucidate how prevalent anurans communicate via chemosignals, and the interaction among multiple sensory modules in this group.

## Methods

### Animals

For the structural and ultrastructural examinations, two male *N. pleuraden* were collected at the Caohai National Nature Reserve (Bijie, Guizhou) in a night of late June, 2017. For behaviour tests, in total 15 male and 40 female *N. pleuraden* were captured at the reserve in nights during June 24th to July 3th, 2018. There were 5 sets of ethological trials conducting sequentially. Animals were collected and maintained separately in lidded plastic boxes (15 × 10 × 6 cm^3^) with moist tissue paper inside on experimental days’ eves, and released back to the field (except for the stimuli providers) after a certain set of trials have been accomplished the next night. Considering this sampling-experimenting-releasing arrangement, the possibility that identical individual was introduced to more than one set of trials could not be ruled out.

All the *N. pleuraden* used in this study were sexually mature as we located the male frogs via their advertisement calls and the females were larger than 55 mm in snout-vent length with swollen abdomens (an indication of gravidity). Seven males (two in 2017 & five in 2018) were euthanized by bath immersions in overdosed (3 g/L) tricaine methanesulfonate (MS-222) solution for 20 min and then washed by distilled water before skin or gland excisions.

Animal collection and experiment permissions were issued by the Management Office of the Caohai Nature Reserve (CHNR2017062101 & CHNR2017062301) for this study and all experimental procedures involving animal attendances strictly followed the regulation formulated by the Animal Care and Use Committee of Chengdu Institute of Biology, CAS.

### Structure and histochemistry

Dorsal skin patches (about 16–25 mm^2^) and the postaxillary glands (about 1 cm^2^) were stripped from the two euthanized male *N. pleuraden*, and the widths, heights and thicknesses of the postaxillary glands were measured. Samples for light microscopy (LM) were fixed in Bouin’s fluid for 24 h, after which they were rinsed in running water for 30 min and immersed in 70% ethanol for another 30 min for discoloration. Dehydration was achieved in ascending ethanol series (80, 95, 100% two changes). Then the samples were cleared in xylene, embedded in paraffin and sectioned transversely at 6 μm using a rotary microtome (RM2235, Leica, Wetzlar, Germany). Sections were stained with: (1) hematoxylin-eosin (HE) for general cytology and histology; (2) combined Alcian blue (pH 2.5) and periodic acid-Schiff (AB-PAS) for acid mucopolysaccharides, sialic acids, neutral carbohydrates and glycoproteins; (3) Masson's trichrome method for collagen fibers and myofibers. The staining procedures followed the instructions in corresponding stain kits (Solarbio Science & Technology, Beijing, China). Stained sections were sealed on microslides with resinene, examined and photographed using a Nikon E200 microscope equipped with an industrial digital camera (18MP 1/2.3″ Color USB3.0, APTINA CMOS Sensor, San Jose, USA). The thicknesses of the epidermal layers in the dorsal skin regions and the postaxillary glands, as well as the maximum heights of simple alveolar glands and inner secretory cells were measured using ImageView (www.microdemo.com/download/imageview). Values represent mean ± standard error consistently. In dorsal skin and the postaxillary gland sections, we referred different simple alveolar gland types as ordinary serous glands (OSG), type I mucous glands (I MG), type II mucous glands (II MG) and specialized mucous glands (SMG) according to widely accepted preexisting terminologies [1, 22, 50, 51].

### Ultrastructure

For scanning electron microscopy (SEM), the postaxillary glands samples (about 25 mm^2^) were fixed in 3% glutaraldehyde (6 h, 4 °C), then rinsed in 0.1 M Sorensen phosphate buffer (pH 7.2) for 3 times (15 min each time) and postfixed for 1 h in 1% osmium tetroxide in the same buffer. The samples were again rinsed and dehydrated through a graded series of ethanol (30, 50, 70, 80, 90, 100%, 30 min for each grade and samples were cut into small pieces with a razor blade in 70% ethanol). After the immersion in isoamyl acetate to displace ethanol, the samples were mounted on aluminium stubs, dried in a critical point apparatus (K850, Quorum Technologies, East Sussex, UK), coated with gold palladium in a sputtering device (E-1045, Hitachi, Tokyo, Japan), and observed in a scanning electron microscope (SU3500, Hitachi) at 15 kV.

For transmission electron microscopy (TEM), glands samples were fixed, postfixed and dehydrated following the same procedure in SEM sample preparation. After dehydration, the samples were cleared in propylene oxide and embedded in epoxy resin. Semithin (1 μm) and ultrathin (70 nm) sections were cut using a ultramicrotome (EM UC7, Leica) and diamond knives (Ultre 35°, DiATOME, Biel, Switzerland). Semithin sections were stained with 1% toluidine blue in 1% sodium carbonate and examined under a light microscope for preliminary gland localization. Ultrathin sections were mounted on uncoated 200 mesh copper grids and electron-dense stained with uranyl acetate (25 mg/ml), followed by lead citrate (2 mg/ml). Grids were viewed using a transmission electron microscope (Tecnai G^2^ F20, FEI, Hillsboro, USA), operating at 60 kV and photographed using an ES1000W Erlanshen CCD Gatan digital camera (Gatan Inc., Pleasanton, USA). Spliced figures of structural and ultrastructural observations’ results were prepared using Adobe Photoshop CS6 (www.adobe.com/Photoshop).

### Stimuli

To perform animal preference tests, we prepared both chemical and auditory stimuli. As for chemotaxis detections, the two bilateral postaxillary glands (weight about 0.14 g) or a dorsal skin patch of the same weight were excised from a euthanized male *N. pleuraden* and wiped away blood gently with wet tissue paper, then suspended in 5 ml cold (4 °C) distilled water and homogenized using a glass tissue homogenizer. Cold distilled water was added to the homogenate to make the final volume 10 ml. After a 30 s shaking, the homogenate was divided into 10 aliquots (1 ml each), which were stored at 4 °C and used on the night of the preparing day. Each aliquot contained the equivalent of one fifth of one postaxillary gland or a tenth of a removed dorsal skin patch. When used as chemical stimulus, one aliquot was injected into a sponge block (1 cm^2^), and then the sponge block was attached to one end of the experimental tank (below water level). Sponge blocks injected with distilled water, postaxillary gland aliquot mixed with trypsin solution (Trypsin lyophilized powder (Sigma-Aldrich, St. Louis, USA) dissolved in 0.9% sodium chloride to bring the concentration to 25 g/L, added 1 ml into the aliquot and gently shook for 5 min at ambient temperature) and distilled water mixed with trypsin solution were also placed in the tank as optional stimuli for tested frogs.

The advertisement calls of male frogs were recorded using a solid state recorder (PMD661 MK II, 16bit, 44.1 kHz, Marantz, Kawasaki Kanagawa, Japan), and the connected microphone (ME66 with K6 power module, Sennheiser, Wedemark, Germany) was pointed to calling frogs in close vicinity. A representative recording consisting of six calls (three notes per call) in which the call duration, the fundamental frequency and the dominant frequency were 0.70 ± 0.01 s, 539.04 ± 16.83 Hz and 1874.75 ± 19.93 Hz, respectively, was loop played to tested subjects via two portable speakers (Clip+, JBL by Harman, Stamford, USA) wired to two laptops and positioned equidistantly to the center of the experimental tank at the opposite ends. The playbacks were set to present non-simultaneously through the two speakers, with 20 s delay in one speaker. Before the introduction of tested animal in behavioral trials that involved acoustical stimuli, calls from the two speakers were adjusted to 54 dBC SPL (measured at the center point of the experimental tank using AWA6291 acoustimeter, Aihua, Hangzhou, China). The visualization of frog advertisement calls (see Additional file 1, Fig. S3) was fulfilled using Praat (version 6.1.06, www.praat.org).

### Preference tests

There were five sets of behavioral tests, each composed of 10 trials and designed to evaluate the responses of tested frogs of one sex toward two stimuli (or two stimuli combinations). The experimental tank was self-made: a 1 m polyvinyl chloride rain gutter with rectangular cross section (18 cm wide) was sealed at both ends using matched plastic lids. Markers were painted on the edge of the tank with black marker pen to subdivide it into 3 areas: from the center point to extend 10 cm bi-directionally was assigned as the waiting zone, whereas the two areas (40 × 18 cm^2^) at both ends were referred as the selected zones (see Additional file 1, Fig. S4). A network surveillance camera with infrared module (PE2200, Panbao Technology, Shenzhen, China) was set up above the tank and manipulated by the software IPClient to record animal behavior.

The general procedure of a single trial was described here: Aged tap water was added to the experimental tank to bring the depth to 6 cm, a tested frog was then placed at the center of the waiting zone with a wire-mesh box covered above to restrain its movement. At meantime, two different stimuli were introduced to two different ends of the tank based upon random side assignments and video recording started. After 10 min acclimation, the wire-mesh box was removed, thus the tested animal was allowed to move freely in the tank. Once the tested frog moved out of the waiting zone, 10 min timing was initiated, after which the frog was collected out from the tank, so as the stimuli, the camera was switched off, then the tank was cleaned thoroughly with detergent and rinsed by aged tape water for several times.

The five sets of trials concerning different conjectures were: (1) To test whether the postaxillary gland secrete sexual attractants: female chosen between the homogenate of postaxillary glands and distilled water; (2) To test whether the postaxillary gland’s secretions are intrasexual attraction or repellent: male chosen between the homogenate of postaxillary glands and distilled water; (3) To test whether male dorsal skin secrete female attractants: female chosen between the homogenates of male dorsal skin and the distilled water; (4) To test how auditory signals interact with chemosignals: female chosen between the homogenate of postaxillary glands and distilled water, when male advertisement calls were presented in both ends of the experimental tank; (5) To reveal nature of the potential sexual attractants released by the postaxillary gland: female chosen between the homogenate of postaxillary glands added trypsin solution and distilled water added trypsin solution. Trials were conducted between 20:00 and 02:00 when frogs are active. The yard of a famer house (26.873391°N, 104.225566°E, 2178.84 m asl.) near the Caohai Nature Reserve was rented to execute behavioral tests, so the environmental factors were similar to the animals’ natural breeding sites. The five series of trials were initiated on June 25th, June 27th, June 29th, July 1st and July 4th, respectively. The mean air, water temperature and humidity were measured as 20.0 ± 0.3 °C (18.5–21.8 °C), 20.2 ± 0.2 °C (18.8–21.1 °C) and 75.6 ± 1.1% (70.9–82.8%) respectively.

### Video analyses and statistics

Recorded behavioral videos were analyzed using the embedded player of IPClient. Timing began at the point when a tested frog left the waiting zone, and the total time that a frog spent on staying at each stimulus side was calculated (use the midline of the experiment tank as side switch sign). Two series of duration time data obtained from one set of preference trials were compared. First, normality of the distribution was examined using Shapiro-Wilk test. If the data were normally distributed, homogeneity of variances was checked through Levene’s test. Pooled t-test was used for variances-equal data, otherwise the t’-test was applied. When normality test failed, the Wilcoxon rank-sum test was introduced. The epidermis’ thicknesses of the dorsal skins and the postaxillary glands (*n* = 10 for each position) were also compared following the above procedure.

Heights of the secretory cells in the three kinds of mucous glands (I MG, II MG and SMG, *n* = 30 for each gland type) were analyzed using one-way ANOVA followed with the Bonferroni-adjusted least significant difference (Bonferroni-adjusted LSD) test, after the variables had been confirmed to possess normal distributions and equal variances. The same analyzing procedure was brought to compare the heights of the 4 types of gland (3 mucous types plus OSG, *n* = 10 for each type).

All tests were two-tailed with a significance level (α) of 0.05 and performed in SAS 9.3 (SAS institute Inc., Cary, USA).

## Supplementary information

**Additional file 1. **Basic information on *Nidirana pleuraden* and ethological experiment equipment. Supplementary figures show the regression of the postaxillary gland out the breeding season of *N. pleuraden*, the animals’ breeding sites, waveform and spectrogram of the male advertisement call, and set-up of the animal preference test.

## Data Availability

All data used and/or analyzed during the current study are available from the corresponding author on reasonable request.

## Abbreviations

I MG: Type I mucous gland
II MG: Type II mucous gland
AB: Alcian blue
ANOVA: Analysis of variance
HE: Hematoxylin-eosin
LM: Light microscopy
OMG: Ordinary mucous gland
OSG: Ordinary serous gland
PAS: Periodic acid-Schiff
SDSG: Sexually dimorphic skin gland
SEM: Scanning electron microscopy
SMG: Specialized mucous gland
SSG: Specialized serous gland
TEM: Transmission electron microscopy
