# Supplementary material for: Structural and bio-functional assessment of the postaxillary gland in Nidirana pleuraden (Amphibia: Anura: Ranidae)
Source: Zoological Lett. 2020 Jun 5;6:7. doi: 10.1186/s40851-020-00160-w (PMC7275488; doi:10.1186/s40851-020-00160-w)
Supplement: Supplementary file 1 — Additional file 1. Basic information on Nidirana pleuraden and ethological experiment equipment. Supplementary figures show the regression of the postaxillary gland out the breeding season of N. pleuraden, the animals’ breeding sites, waveform and spectrogram of the male advertisement call, and set-up of the animal preference test. [file 40851_2020_160_MOESM1_ESM.docx]

**Additional File 1**

**Structural and bio-functional assessment of the postaxillary gland in *Nidirana pleuraden* (Amphibia: Anura: Ranidae)**

Yuzhou Gong, Yiwei Zeng, Puyang Zheng, Xun Liao, Feng Xie^*^

*Correspondence: xiefeng@cib.ac.cn


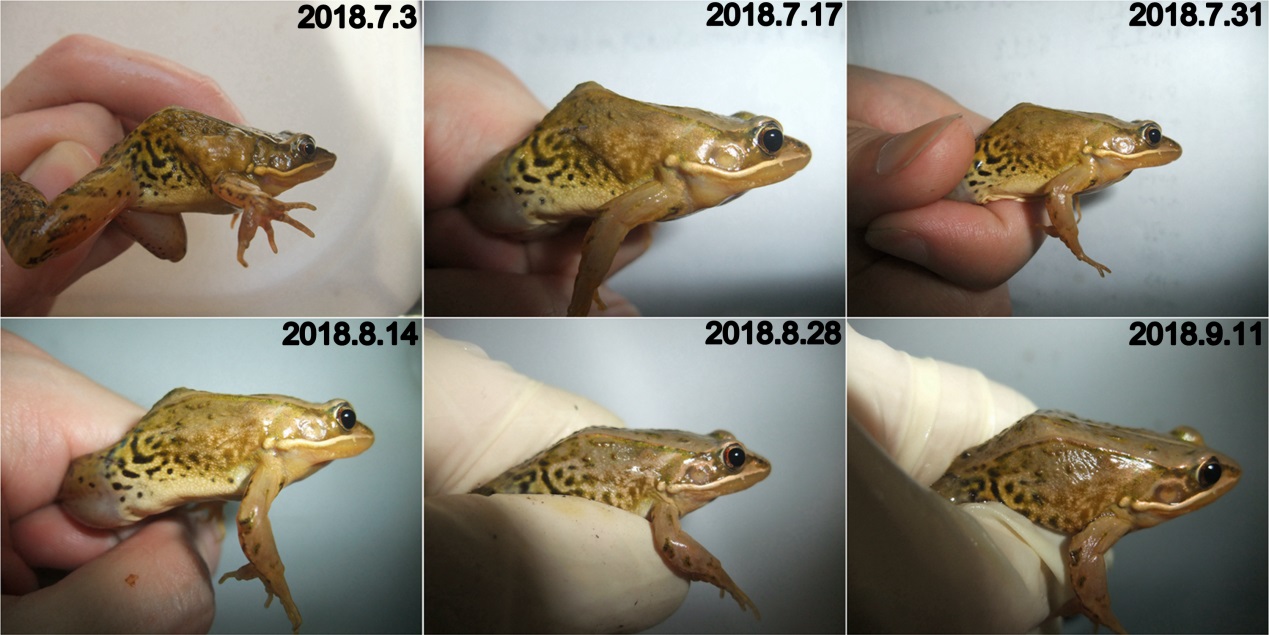


Fig. S1 Regression of the postaxillary gland in male *N. pleuraden* after the breeding season (June to July). The date of photographing is indicated in the upper right corner of each panel.


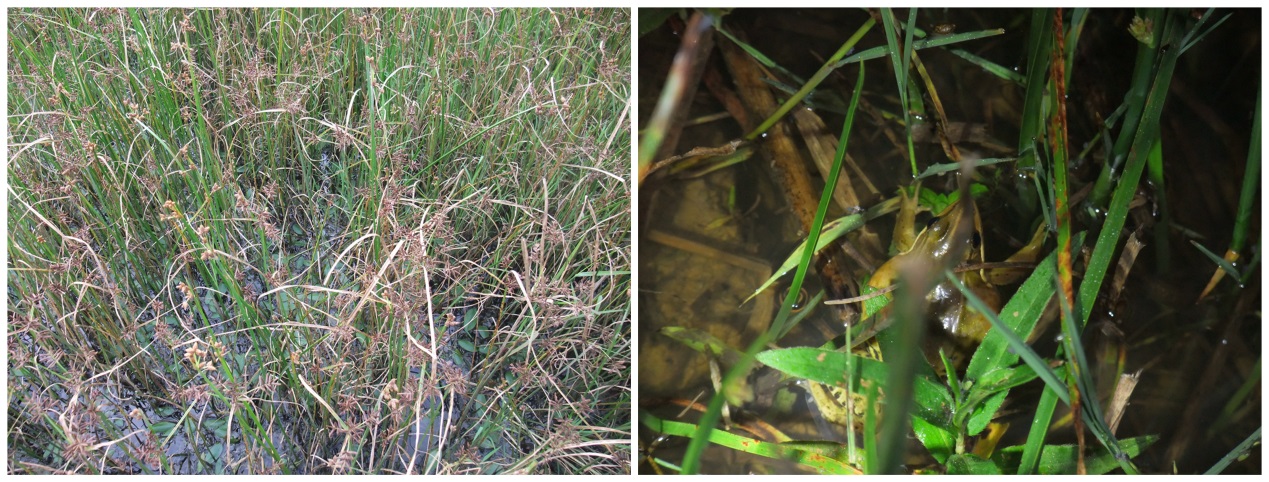


Fig. S2 Breeding sites of *N. pleuraden* are lush in hydrophytes and a male frog calls in such covert environment.


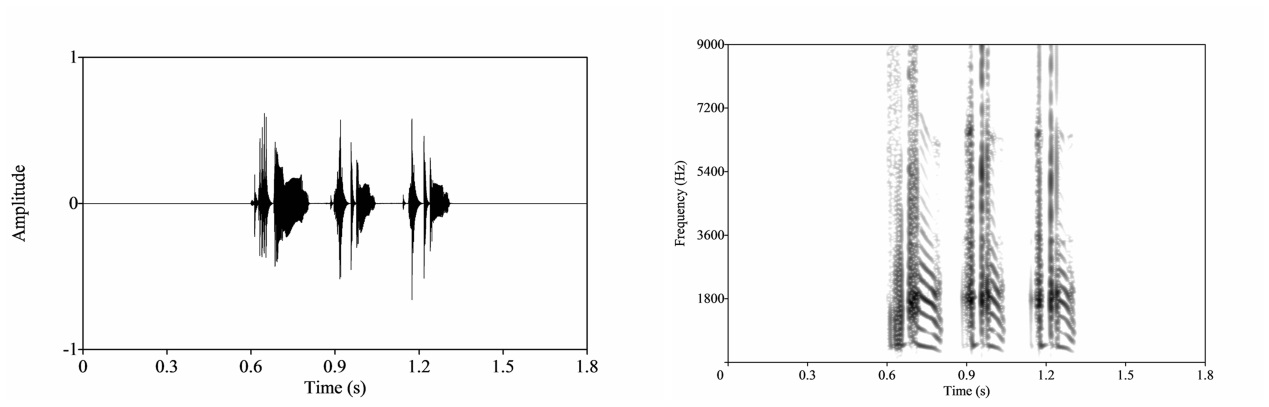


Fig. S3 Waveform (left) and spectrogram (right) of the male advertisement call used as auditory stimulus. The average call duration is 0.70 s, with the fundamental frequency and dominant frequency are 539.04 Hz and 1874.75 Hz, respectively.


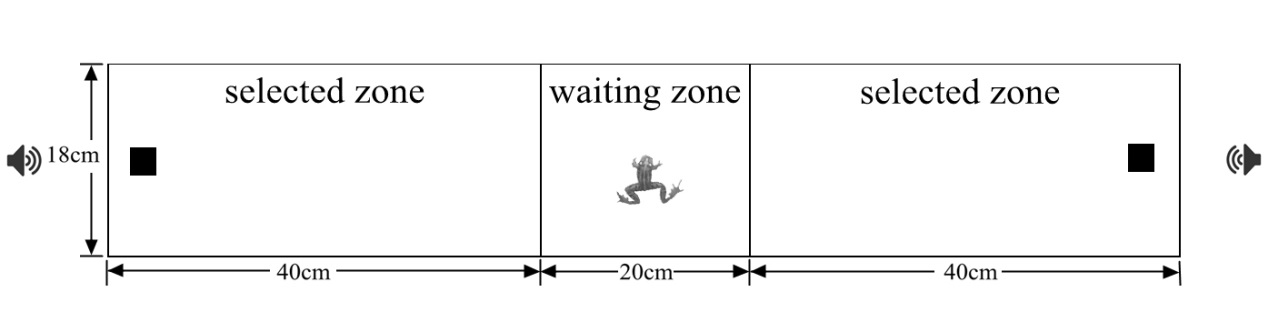


Fig. S4 Diagram of the setup of animal preference trials. Black boxes at the tank ends represent chemical stimuli, and speakers only work in trial series 4 with an intention to explore the interaction between chemosignal and sound.
